# Factors Influencing Kidney Transplantation Experiences for Patients From Culturally and Linguistically Diverse Backgrounds: A Qualitative Study

**DOI:** 10.1111/hex.14166

**Published:** 2024-08-03

**Authors:** Kimberley Crawford, Catherine Wilson, William R. Mulley, Nigel D. Toussaint, Elaine Kennedy, Narissa Andrew, Andrea Ward, Mandy Truong

**Affiliations:** ^1^ Monash Nursing and Midwifery Monash University Clayton Victoria Australia; ^2^ Nursing Institute of Health and Wellbeing Federation University Mount Helen Victoria Australia; ^3^ Department of Nephrology Monash Medical Centre Clayton Victoria Australia; ^4^ Centre for Inflammatory Diseases, Department of Medicine Monash University Clayton Victoria Australia; ^5^ Department of Nephrology The Royal Melbourne Hospital Parkville Victoria Australia; ^6^ Department of Medicine (RMH) University of Melbourne Parkville Victoria Australia

**Keywords:** chronic kidney disease, culturally and linguistically diverse background, kidney transplantation, racial and ethnic minorities

## Abstract

**Background:**

Disparities in aspects of chronic kidney disease progression and management exist for patients from culturally and linguistically diverse (CALD) backgrounds, including with treatment and outcomes for kidney transplantation.

**Objective:**

This study aimed to explore factors that impact kidney transplant outcomes from the perspective of kidney transplant recipients (KTRs) from CALD backgrounds and their family caregivers.

**Methods:**

A descriptive qualitative design was utilised. Participants were recruited from two tertiary hospitals in Victoria, Australia. Semi‐structured interviews were conducted with KTRs who were born overseas in countries where English is not the primary language. Interviews were also conducted with family caregivers. Analysis was guided by the Framework Method, and emergent subcategories were mapped into the categories identified in Andersen's Health Service Utilisation Model.

**Results:**

Data from 21 KTRs and five caregivers were grouped under the categories of Population Characteristics, Environment, Health Behaviour and Outcomes. KTRs believed that neither culture nor religious beliefs impacted how they managed their transplant or healthcare utilisation. KTRs expressed satisfaction with their care, felt no inequity with how they were treated by health professionals and expressed gratitude for the Australian healthcare system. Language did not necessarily impact transplant outcomes, but there was a reliance on interpreters for non‐English‐speaking patients as most written information was in English. Caregivers were instrumental in providing support but discussed the challenges involved.

**Conclusion:**

This study explored factors influencing kidney transplantation for KTRs from a CALD background. The study provided insight into how to deliver quality healthcare to these patients, highlighting the importance of health services providing information that is written in the patient's own language and respectively asking KTRs about their health beliefs or customs. Caregivers were instrumental in supporting KTRs, but there is a need to better prepare them for this role.

**Patient or Public Contribution:**

Patient and public involvement was integrated into the design and delivery of the study. KTRs from CALD backgrounds assisted with framing the research questions and offering advice on the recruitment and data collection process.

## 1 Introduction

Chronic kidney disease (CKD) is a progressive condition characterised by structural and functional changes to the kidneys due to various causes [1]. CKD impacts between 8% and 16% of the population worldwide [2]; however, the incidence, prevalence and progression of CKD do vary within countries by ethnicity and social class [3]. In a small proportion of cases, progressive CKD leads to kidney failure, where treatment by dialysis or a kidney transplant is required [4].

CKD is of particular interest in the study of health inequalities as there is a marked social gradient in the incidence of the disease [5]. Disparities by racial and ethnic minority status exist around the world where individuals from culturally and linguistically diverse (CALD) backgrounds bear a disproportionate burden of CKD and have reduced access to kidney transplantation [6, 7, 8]. A scoping review has identified disparities for individuals from CALD backgrounds in all aspects of kidney disease progression and management, including the transplantation process and transplantation outcomes [9]. When people from CALD backgrounds experience kidney failure, they take longer to be wait‐listed for a transplant. As an example, Asian and Black minority groups had reduced access to pre‐emptive listing in the United Kingdom [10]. Although individuals from CALD backgrounds were disproportionately represented in populations with kidney failure, these individuals were found to receive fewer kidney transplants [9]. In the United States, Black people with kidney failure were more than 50% less likely to receive a living donor transplant [11]. Research also indicates that kidney transplant outcomes for individuals from CALD backgrounds may be less favourable. These individuals are more likely to experience delayed graft function, reduced graft survival [12], are more likely to experience 5‐year graft loss [13], and cultural status was identified as a risk factor for medication non‐adherence [14]. People from a CALD background are a heterogeneous group with different languages, cultures, religions, social values and migration trajectories, which can impact health outcomes [15].

These previous studies were quantitative. There is a lack of qualitative studies contributing to the evidence base, which can provide a deeper understanding of healthcare use and delivery. Further research exploring individual, cultural and social factors impacting people with kidney failure from a CALD background may help to understand and address why these disparities exist.

This study aimed to explore factors that impact kidney transplant outcomes from the perspective of kidney transplant recipients (KTRs) from CALD backgrounds and their family caregivers.

## 2 Methods

### 2.1 Study Design

A descriptive qualitative design was employed [16]. The study is reported according to the Consolidated Criteria for Reporting Qualitative Research (COREQ) guidelines [17]. Two tertiary health services that specialise in adult kidney transplantation in Victoria, Australia participated in the project.

### 2.2 Conceptual Framework

The conceptual framework underpinning this study is Andersen's Behavioural Model of Health Services [18]. This framework has been widely used in research investigating health service use across a variety of health conditions and healthcare settings [19]. The framework has been previously applied in nephrology research and studies exploring health service use, including racial and ethnic minority groups [20, 21, 22, 23].

### 2.3 Study Context

In Victoria, where the study was conducted, 30.2% of the population speak a language other than English at home [24]. Across the Australian population, there are a range of cultural values and beliefs that have the potential to influence illness experiences, access to care and interactions with healthcare providers [25]. Australia has a universal health coverage system, Medicare, that provides free public hospital care for all Australians. The scheme also subsidises a range of other services outside of the hospital and prescription medication [26].

### 2.4 Study Participants

KTRs from a CALD background, defined as someone born in a country other than Australia in which the official language is not English [27], were eligible to participate. Any Victorian resident receiving care from the participating hospitals over 18 years of age could be included. There were no exclusions based on the length of time living in Australia or the primary language spoken. Family caregivers of current or past KTRs from a CALD background with knowledge of the transplant recipients' condition were eligible to participate. There was no limit on country of birth or language spoken or relationship to the KTR. The family member did not need to be from a CALD background.

### 2.5 Recruitment

A mix of purposive and convenience sampling was used to recruit participants. Recruitment was undertaken by health professionals at each participating hospital. Health professionals identified KTRs who met the eligibility criteria and spoke to them over the telephone. Potential KTRs and caregivers were also approached during clinic appointments. Contact details of those who expressed an interest were forwarded to the research team. For non‐English‐speaking participants, an interpreter assisted with recruitment. No records were kept of the number of potential participants who declined to participate.

### 2.6 Data Collection

Interviews were conducted using a semi‐structured interview guide (Table 1), initially developed by the research team and reviewed by the patient consumer group. Interviews were conducted from April 2021 to September 2021. All interviews were undertaken over the telephone or via video conferencing, in accordance with participants' preference. Two PhD‐qualified researchers (K. C. and M. T.), with qualitative research experience, conducted the interviews and neither had a prior relationship with the participants. M. T. is a public health researcher with a South‐East Asian background with experience conducting research with people from CALD backgrounds. K. C. is from an Anglo‐Australian background and has experience conducting research with KTRs and caregivers. Professional interpreters assisted with all interviews with non‐English‐speaking participants and provided word‐by‐word interpretation. Interviews with KTRs continued until no new information emerged. Researchers attempted to recruit more caregivers but were unsuccessful. Demographic information was collected from all participants. Participants were given the option to review their transcripts before data analysis, but no participants accepted this offer.

**Table 1:** Interview guide.

Kidney transplant recipients
‐ Can you tell me about the care you have been receiving from the hospital? ‐ Can you explain how the healthcare team involve you in the decisions about your care? ‐ Does anyone help you get to your appointments or during your appointments? ‐ What are some positive/negative things about going to the renal clinic or the care you receive? ‐ Since your transplant, how has your life changed? ‐ Thinking back to when you were waiting to receive a transplant, what was your experience like? ‐ What are your perceptions of life post‐transplantation? ‐ What's been the hardest thing about getting a transplant? ‐ Do you feel comfortable with the information that is provided to you by your healthcare team? ‐ What advice would you give the doctors or nurses so they can help the next person getting a kidney transplant? ‐ Are there any cultural traditions that you follow? What impact would these traditions have on your kidney transplant?
Caregivers
‐ What kinds of things did you do for your family members in relation to their kidney transplant? ‐ What has been the hardest thing about caring for or supporting your family members? ‐ Did you have any support yourself during this time? ‐ What do you think of the doctors and nurses at the hospital who assist your family members with managing their transplant? ‐ Can you explain how the healthcare team involve your family members in the decisions about their care? ‐ Do you think you are kept well informed? ‐ What advice would you tell the doctors or nurses so they can better help future kidney transplant patients? ‐ What advice or knowledge do you have for other family caregivers for kidney transplant patients? ‐ Are there any cultural traditions that <name of recipient> follows? What impact would these traditions have on <name of recipient> kidney transplant?

### 2.7 Data Analysis

Interviews were audio‐recorded and then professionally transcribed verbatim. NVivo software (version 20; QSR International) was used to manage and process data. Analysis was undertaken by three researchers (M. T., K. C. and C. W.). C. W. has over 25 years of CKD experience that spans clinical, education and management. The analysis was guided by the Framework Method [28]; each researcher independently coded the same four interview transcripts. The researchers met and agreed on the codes to apply to subsequent transcripts and the initial categories were developed. All data were charted into a framework matrix, with a summary of the data and key quotes. The subcategories were then mapped onto the categories identified in Andersen's Health Service Utilisation Model. Researchers met regularly to discuss the evolving data analysis and to ensure the credibility of the study. Including information from the KTR and their family ensured data triangulation and detailed records of all stages of the research study were retained to ensure dependability.

### 2.8 Patient and Public Involvement (PPI)

PPI was integrated into the design and delivery of the study. Three KTRs from a CALD background, which were reported as Greek, Japanese and Sri Lankan assisted with the project. As the study was initially being developed, researchers consulted with the three patient consumers individually over the telephone to seek feedback on the potential recruitment process and the data collection process. When the interview guides were being developed, patient consumers suggested potential questions and reviewed the final interview guides for appropriateness.

### 2.9 Ethics

Both participating hospital ethics committees and the university provided ethics approval (Monash Health HREC reference: 64705). Participants were informed that participation was voluntary and confidential. Verbal consent was collected and recorded by the researchers. For non‐English‐speaking participants, consent was collected with the assistance of an interpreter and where possible study information was translated into the participant's preferred language.

## 3 Results

**Figure 1:**
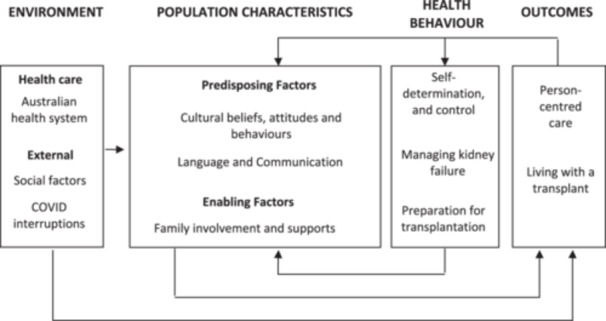
Summary of categories and subcategories influencing transplantation outcomes for kidney transplant recipients from a racial/ethnic minority in accordance with Andersen's Behavioural Model of Health Service.

Experiences from 21 KTRs and five caregivers offered unique, converging and complementary perspectives. These were grouped into the following categories: Population Characteristics, Environment, Health Behaviour and Outcomes within Andersen's Health Service Utilisation Model (Figure 1).

Participant demographics are presented in Table 2. There was significant cultural, linguistic and religious diversity among the KTRs. The study included four KTR and caregiver dyads, each of which was interviewed.

**Table 2:** Participant demographics.

Characteristics	Total
**Kidney transplant recipients**	***n* = 21**
Male [*n* (%)]	15 (71)
Age, years [Median (IQR)]	63 (58–66)
Time living in Australia, years [Median (IQR)]	29 (15.5–33.5)
Country of origin [*n* (%)]	
Vietnam	3 (14.2)
Singapore	1 (4.8)
Lebanon	1 (4.8)
Sri Lanka	2 (9.5)
Philippines	2 (9.5)
Sierra Leone	1 (4.8)
Ethiopia	2 (9.5)
China	3 (14.2)
Cambodia	2 (9.5)
Afghanistan	1 (4.8)
Serbia	1 (4.8)
Iran	1 (4.8)
India	1 (4.8)
Time since transplant, years [Median (IQR)]	2 (0.75–3.0)
Required an interpreter [*n* (%)]	7 (33)
Faith/religion [*n* (%)]	
Taoism	1 (4.8)
Buddhism	3 (14.2)
Catholic	1 (4.8)
Christian	5 (23.9)
Muslim	3 (14.2)
Hindu	1 (4.8)
None	7 (33.3)
**Caregivers**	***n* = 5**
Male [*n* (%)]	2 (40)
Age, years [Median (IQR)]	50.5 (39–58)
Born in Australia [*n* (%)]	1 (20)
Required an interpreter [*n* (%)]	1 (20)
Relationship to kidney transplant recipient [*n* (%)]	
Wife	2 (40)
Husband	1 (20)
Daughter	1 (20)
Son	1 (20)

Abbreviation: IQR, interquartile range, reported as quartile 1 and quartile 3.

### 3.1 Population Characteristics

#### 3.1.1 Cultural Beliefs, Attitudes and Behaviours

Under Andersen's Health Service Utilisation Model, predisposing factors are characteristics intrinsic to the individual including cultural beliefs, attitudes and behaviours. Many KTRs felt that their cultural or religious beliefs and traditions did not impact how they managed their transplant. One example is that some KTRs with a faith, where they are normally required to observe a month of fasting (i.e., Ramadan), explained that they were not required to fast during this period nor did they fast.

In Islam, you don't have to do anything that is harmful for you. They say that if you know that fasting is harmful to your health, it becomes prohibited.

(KTR#13)

Many KTRs spoke of being committed to their faith/religion and saw that their faith guided their decision‐making on personal life matters but trusted medical advice when it came to managing their care. One KTR from a Chinese immigrant background described how he had to overcome his trust and reliance in traditional Chinese medicine to accept his diagnosis of kidney failure and follow Western medicine:

I think the biggest roadblock was the fact that the trust level with the TCM [Traditional Chinese Medicine] was so high that you don't even question that. Because in your mind, when you talk about TCM … you're talking about a practice that goes back thousands of years, the Chinese traditional medicine, thousands of years. And very few of us understood the way in which the science of the western medication is being handled.

(KTR#1)

For many, there had been years since diagnosis of kidney disease to the time they received their kidney transplant, and they had already received treatment by dialysis, so they were acquiescent to Western medical treatment. Others spoke of living in Australia for so long that they had acculturated more to Australian culture than to their heritage culture:

My culture, I forgot already my culture. I live here longer than in my country, so I adapt Australian life than in the Philippines because I live only the Philippines 25 years. I cannot really see that affects my culture.

(KTR#5)

Caregivers echoed these types of sentiments, and they believed that neither the culture nor religious beliefs of their loved ones affected how they managed their transplant. One son with Chinese heritage didn't see that his family had any traditional or cultural differences that were too different from Australia and his father (KTR#9) didn't do anything different to Western culture.

#### 3.1.2 Language and Communication

Language and communication were predisposing factors. KTRs explained the challenges in understanding the medical jargon as a layperson. It was common for KTRs to indicate that they had understood health professionals rather than seek further clarification:

The hardest part of the transplant, I should say, it is English. Because after all, English is not my mother tongue. Chinese is … I don't really fully understand. And sometimes with new medication I would check with the doctor. Sometimes I would just let it go and I didn't really ask.

(KTR#8)

The additional challenge for non‐English‐speaking KTRs was that all educational material was in English. Most non‐English‐speaking KTR were reliant on verbal education from health professionals through an interpreter, mostly professional interpreters. Although most non‐English‐speaking KTRs reported no issues with interacting with the health professionals, as one KTR reported, it was still an obstacle they needed to overcome:

The most difficult part is the language barrier. I think it's just the communication, and the passing on of information that's hard.

(KTR#11)

Some had learnt terms related to the management of their kidney transplant so they could converse with health professionals without an interpreter. Caregivers expressed concerns that medical information could not be translated with complete accuracy through an interpreter:

I would find a way to get the doctors to break it a bit finer and in a way that I can understand it and in a way that I can translate to my dad as well. Translation is pretty hard because sometimes, even though when you know what it means in English, I may not know what it is in Chinese.

(Caregiver#5)

#### 3.1.3 Family Involvement and Supports

Enabling factors are resources or tools that encourage healthcare usage. Every KTR spoke of the support network that had assisted their transplant journey, which included spouses, children, siblings, cousins and grandchildren. This was in the form of personal support or moral support. Some family members provided direct assistance with managing the KTR's care or were used as the interpreter and were the point of contact for the hospital. One non‐English‐speaking KTR admitted that he relied on his grandchild:

She [granddaughter] does almost everything for me. For example, I'm illiterate, so I don't know what to do, but she reads the instructions very carefully. Each night prepares my medication and also provides all other assistance, like, with food and with other—washing my clothes—everything.

(KTR#13)

If the KTR had no extended family in Australia, the spouse was solely responsible for providing support, which was demanding. The caregivers reiterated the personal and medical support they provided for their loved ones. One caregiver spoke of the emotional ‘rollercoaster’ in the initial months post‐transplantation:

I have to admit it's been hard; it's been frustrating … And when she has a bad day, it makes you feel bad as well. You try and do your best to make sure she's feeling okay.

(Caregiver#1)

This caregiver was able to seek support from a friend but admitted that when his wife (KTR#6) was struggling, he didn't know how to help or who to contact for assistance. This feeling of stress or burden was common for most caregivers. One wife explained her husband (KTR#1) struggled with his mental health during treatment by dialysis and recommended the hospital needed to prepare the caregivers:

I'm just very grateful now because my last five years were miserable. No one can understand what I had to go through … I was in tears most of the time. I think if someone could prepare some sort of a document to prepare the caregiver mentally of what's to come from the patient, from the physical aspects as well as the mental aspects.

(Caregiver#2)

### 3.2 Environment

#### 3.2.1 Australian Health System

Many KTRs compared the Australian health system to the health system in their country of origin, which often requires out‐of‐pocket payment for any healthcare received. This cost had been a barrier to them visiting the hospital or doctor, so instead, some were reliant on herbal medicine. Those who had experience with the health system in their country of origin reported distrust of the system and felt doctors were not always honest. In comparison, KTRs praised the Australian health system, the expertise of the health professionals and the facilities. Many were grateful to have received a transplant, with some realising that transplantation may not have been possible in their country of origin. One KTR explained that in Australia he felt that all patients were treated equally:

The issue is that, in Iran, they are so fanatic that they do not give importance to somebody who is poor or somebody who is a stranger or from another country, but in here, it is not—people are respected. The poor people are even respected.

(KTR#13)

This equity also translated to who had access to the available transplants and the fairness of this process, as one participant explains:

I believe that the system is fair enough and I also think that there is no sort of preference of race or all the preference in conducting with transplants … Fair procedures, fair decision making.

(KTR#10)

One caregiver was acutely aware that her husband (KTR#1) might have experienced a very different outcome had they remained in their country of origin:

If we had stayed in Singapore, trust me, we would either have gone bankrupt just paying for dialysis or he would already have died by now. I feel very, very grateful and I think the system is very good … In Singapore, there is this saying, “When you are diagnosed with kidney failure, you're doomed to die.”

(Caregiver#2)

#### 3.2.2 Social Factors

There were social factors that impacted KTRs such as the adequacy of their living arrangements, issues with family relationships and challenges getting to the hospital:

I don't have a car; I always use public transport. And, even when I went for operation for transplant kidney I went by bus.

(KTR#2)

A couple of KTRs were dependent on Government payments to be able to obtain their medication. Another explained when he initially came to Australia as a skilled migrant and was diagnosed with kidney failure, he was denied support from the Government:

I had to be here two years to be able to get any benefit from Centrelink [Government Agency]. So at that point there was no support, so that was a big struggle going through dialysis and being identified as a kidney patient and no support from the government.

(KTR#16)

Another spoke of losing his business when he was initially diagnosed with kidney disease, with a lack of knowledge of the system to know of potential financial support available to him. One caregiver spoke of her struggles with trying to organise appropriate Government housing for her mother who had recently experienced rejection and returned to dialysis.

#### 3.2.3 COVID‐19 Interruptions

During the COVID‐19 pandemic, face‐to‐face appointments at the hospital ceased, and all KTRs received phone teleconferencing. Most KTRs reported no difference in their care, but one KTR mentioned that it limited the doctor's ability to do a full consultation:

[In person] *doctor check you up on the chest. And look in your face. From the phone, you know, you may be feeling it's all right, but the doctor don't see your face, your eyes*.

(KTR#17)

Non‐English‐speaking patients had access to an interpreter during these phone conversations and felt they were still able to have all their questions answered. Many new KTRs mentioned that the lockdowns gave them an excuse to not have visitors:

Since it was in COVID time, no one is just allowed to come to your house so fortunately, I became safe.

(KTR#7)

### 3.3 Health Behaviour

#### 3.3.1 Self‐Determination and Control

Many KTRs displayed a determination to take control of their healthcare, including managing their medication.

I don't want to take anyone's help. That's all. It's not I don't want to, but if it's something I can do, I will do. And if it is not in my hands, then I will go for help.

(KTR#20)

The initial months post‐transplantation were difficult but as one KTR explained she could never give up:

And if I give up, I give up on life. I've just got to keep going, and keep climbing up the stairs.

(KTR#3)

Some KTRs sought information beyond health professionals, searching Google or joining Facebook groups to exchange information with other KTRs. One KTR explained that this was to help with his understanding and not because he didn't trust the Australian health system or health professionals. Ultimately, KTRs wanted to do everything to ensure they had a successful transplant outcome, as one explained his motto:

“Take your damn pills”, because it's more than just taking pills. It is the whole attitude towards your life.

(KTR#1)

#### 3.3.2 Managing Kidney Failure

Some KTRs ignored their initial diagnosis of CKD or warnings from health professionals, avoiding frequent health checks, delaying commencement of dialysis or refusing a transplant because the participant didn't feel ready. For others, kidney failure came quickly:

The blood centre, on Monday I went to work and they called me straightaway. And the call was so scary because the way they said to me was, “You just take an ambulance from your workplace and go to the hospital … “Your kidneys doesn't look good.”

(KTR#20)

Participants experienced multiple challenges with dialysis: They were unable to work, needed to manage restrictions, became tired of waiting and committed hours to dialysis. During this period, participants mentioned grappling with their mental health:

During the period of the dialysis and the waiting time for the transplant, I lost hope to continue living. I want to end my life. I saw darkness in my future but the hospital saw my situation and asked me to see the psychologist.

(KTR#10)

Caregivers observed their loved ones struggled with dialysis. One caregiver recognised that her husband hated dialysis; it was torture. Interestingly, KTR#1 did not mention these struggles.

#### 3.3.3 Preparation for Transplantation

Hospitals ran information sessions that covered the medications, surgical procedures and life post‐transplantation with KTRs who spoke of their experiences. Many KTRs reported that they felt prepared for transplantation. Some didn't realise the relevancy of the information or completely took in the information until after transplantation and then didn't feel prepared for managing their medication:

I wasn't prepared enough for this; we don't know it until after the surgery when they come and bring you the book and explain and give you the book with all the side effects.

(KTR#6)

These sessions were conducted in English, so for non‐English‐speaking patients some information was missed:

The hospital organised some seminars. They had invited people who had a transplant to speak to us. However, they were speaking English, and I didn't quite understand what they were saying.

(KTR#8)

### 3.4 Outcomes

#### 3.4.1 Living with a Transplant

KTRs described the dramatic changes that came from transplantation, giving them a new lease on life and a feeling of normal again. Transplantation brought a change in mindset, particularly an improvement in mental health. Some challenges came post‐transplantation, with one KTR who lived in fear that she would lose the kidney and return to ‘the machine’ [dialysis] (KTR#2). Many KTRs described how they would do anything to take care of their kidney:

I appreciate it. I'm honoured to have it, and trying my best, and look after it … Life, gift of life. And I have to treasure that.

(KTR#3)

Some conceded that the first months post‐transplantation were difficult; the kidney was not functioning, and managing the medication and multiple clinic appointments was a challenge. One KTR questioned whether it would have been better to stay on dialysis, but a nurse's encouragement kept her motivated. Despite all these challenges, one KTR commented that it was still better than dialysis.

Caregivers spoke of the change they saw in their family member, better quality of life, a positive mindset and a changed person.

#### 3.4.2 Person‐Centred Care

KTRs expressed praise for the nephrology team, acknowledging that health professionals went out of their way to help.

The transplant people they are amazing, very helpful, make you feel that they need to help you, they understand what you're going through no matter what, small or big, they help you.

(KTR#6)

KTRs spoke of being involved in their care and being informed of their care plan. Non‐English‐speaking KTRs were provided access to interpreters and were satisfied with their care. Many KTRs felt that they weren't treated any differently.

I am just a foreigner, they don't see me that I am a foreigner, they are just looking me as family, so they are so interesting, they are so excellent.

(KTR#7)

The caregivers also praised health professionals and confirmed that their family member was treated the same as any other patient at the hospital:

I don't think that there's any prejudice or anything like that from the doctors … I can't think of any nurse or any incident or anything that would suggest that maybe they were prejudice or dad was untreated unfairly.

(Caregiver#5)

## 4 Discussion/Conclusion

This qualitative study sought to explore the factors that impact kidney transplantation for individuals from a CALD background and their families. Understanding KTRs' and family members' experiences may contribute to understanding how to deliver quality healthcare to these patients.

The scoping review by Wilkinson et al. [9], identified disparities between ethnic and non‐ethnic minorities in all aspects of kidney disease progression, and for kidney transplantation specifically this translated to differences in transplant survival outcomes. Although the previous review suggested that inequality exists for ethnic minorities [9], findings from the current study indicate that KTRs did not perceive themselves to be disadvantaged. The KTRs believed that neither their culture nor religion impacted how they managed their transplant. This potentially might insinuate that KTRs would have no worse outcomes than their Australian‐born counterparts. Further research is needed to explore whether outcomes differ based on demographic factors using population‐based studies.

Structural, institutional, systemic and interpersonal racism has been operationalised within kidney transplantation [29]. Within the current study, an overwhelming number of KTRs and their caregivers expressed satisfaction with the health service and health professionals. KTRs felt that the transplantation process was fair, and they were not treated differently due to their cultural background. It is difficult for individual patients to evaluate the equity of their healthcare provision if they are generally satisfied with their care or compare their treatment to what they may have received in their country of origin, which may be a low‐ or middle‐income country. This indicates the limitation of relying on lived experiences to measure equity at the population level. In addition, Australia has a mandatory public insurance scheme (Medicare) that provides healthcare to all citizens, permanent residents and refugees [30]. This accessibility to a range of health services at reduced or no additional cost means that cost was potentially not a barrier for KTRs accessing healthcare, which may help with patient satisfaction.

The current study highlighted that KTRs perceived that neither their cultural traditions nor religion impacted how they managed their transplant, but this is only their perception, and they only know what they have been told. If health professionals do not actively ask specific culturally tailored questions, then the patient may not be aware what they are doing could harm their transplant. Healthcare globally should be tailored to the individual, as there is no one‐size‐fits‐all approach. As outlined in the 2009 Kidney Disease: Improving Global Outcomes (KDIGO) clinical practice guidelines, variations in practice will inevitably and appropriately occur when clinicians consider the needs of individual patients, availability of resources, and limitations of the health service [31]. Health professionals need to ask about the individual's culture, their religion and how the patient manages their care. When health professionals actively facilitate discussions about cultural differences, it has the potential to promote a more inclusive culture in care [32].

Findings from the current study indicate that language barriers could potentially impact kidney transplant outcomes. Health services need to recognise the language diversity of populations and provide culturally sensitive care [33]. For example, in the United States, it is estimated that by 2044, more than half of all Americans are projected to belong to a minority group [34]. Similarly, in the United Kingdom, 14.5% of their citizens were born outside of the United Kingdom [35]. As migration increases worldwide, there will be many instances that require health professionals to care for a patient with whom they do not share the same language [36]. Health services need to provide information that is written in the patient's own language, so there is less reliance on verbal education through an interpreter who is not always professionally trained. As this study highlighted, there was a reliance on family members to help with care or be a translator, which meant an excessive burden was placed upon the caregivers. These family members also don't have adequate training to be a translator, and there are potential ethical or legal issues with family having this role [37]. Providing patients with information in their own language would enable patients to self‐advocate for their own health. Caregivers can be a support for patients, but there is a need to prepare caregivers of what to expect and what role they might play in assisting the KTR.

The strength of this study was that it included participants from a range of cultural backgrounds. This heterogenous group enabled us to gather a greater overview of factors influencing racial and ethnic minorities rather than focusing on one specific ethnic minority group. This enabled the identification of categories that are shared across different ethnic groups. In addition, this study involved a professional interpreter, so experiences were collected from non‐English‐speaking participants, and these are rarely captured in health research. This also ensured that information was translated accurately and not filtered, which could be a concern if using family members as interpreters.

As other researchers have identified, for patients from a CALD background, having the ability to navigate the health system is an essential skill [38]. A limitation of this study was that it included individuals who had successfully navigated hospital processes and the preparation for transplantation to receive a kidney transplant. These participants were not recent migrants to Australia; some had lived in Australia for a long period. These participants may already have some confidence and have had success with managing the hospital system, which may have influenced their satisfaction with the health service. In addition, participants' socio‐economic factors, such as employment or educational level, were not collected. These factors may have impacted healthcare experiences such as access barriers, as lower socio‐economic status can be associated with worse health outcomes [39]. Health professionals assisted with recruitment, which may have introduced some selection bias. Interviews were conducted by researchers who were not involved in the care of the KTRs, so participants would feel comfortable discussing the care they had received. This study was conducted during the COVID‐19 pandemic, and Melbourne where this study was conducted had experienced one of the most stringent and longest lockdowns globally [40], which made recruitment difficult, particularly for caregivers.

In conclusion, KTRs generally expressed satisfaction with their care and felt no inequity in the transplantation process or in how they were treated by health professionals. This study highlights the importance of health professionals being aware and/or willing to learn about the patient's cultural context. There is a need to incorporate culturally responsive care into a broader clinical perspective, so that the patient as a whole person is considered when care is provided. This would also involve considering the information provided to patients whether it is in the patients' own language and culturally appropriate. Finally, the patient and their caregiver should be supported and encouraged to actively participate in the care encounter and share their cultural beliefs, traditions and personal experiences with the health professionals.

## Author Contributions

**Kimberley Crawford:** conceptualisation, funding acquisition, writing–original draft, methodology, formal analysis, project administration, data curation, validation, and investigation. **Catherine Wilson:** conceptualisation, investigation, writing–review and editing, formal analysis, methodology, and funding acquisition. **William R. Mulley:** investigation, writing–review and editing, methodology, conceptualisation, and funding acquisition. **Nigel D. Toussaint:** investigation, methodology, writing–review and editing, and conceptualisation. **Elaine Kennedy:** conceptualisation, investigation, funding acquisition. **Narissa Andrew:** investigation, methodology, writing–review and editing. **Andrea Ward:** investigation, methodology, writing–review and editing. **Mandy Truong:** conceptualisation, investigation, funding acquisition, writing–review and editing, methodology, formal analysis, and validation.

## Consent

A verbal consent protocol was used for the use of participant data for research purposes. This consent procedure was reviewed and approved by Monash Health HREC, approval number 64705, with the decision dated 20 October 2020. Verbal consent was collected from all participants before data collection commenced. Consent was recorded and stored separately from the interview audio. For non‐English‐speaking participants, an interpreter assisted with consent and data collection.

## Conflicts of Interest

The authors declare no conflicts of interest.

## Data Availability

The data that support the findings of this study are not publicly available due to ethical restraints.
